# Prosopagnosia: face blindness and its association with neurological disorders

**DOI:** 10.1093/braincomms/fcae002

**Published:** 2024-01-05

**Authors:** Kennedy A Josephs, Keith A Josephs

**Affiliations:** Department of Neurology, Mayo Clinic, Rochester, MN 55905, USA; Department of Neurology, Mayo Clinic, Rochester, MN 55905, USA

**Keywords:** prosopagnosia, neurodegenerative, MRI, PET, pathology

## Abstract

Loss of facial recognition or prosopagnosia has been well-recognized for over a century. It has been categorized as developmental or acquired depending on whether the onset is in early childhood or beyond, and acquired cases can have degenerative or non-degenerative aetiologies. Prosopagnosia has been linked to involvement of the fusiform gyri, mainly in the right hemisphere. The literature on prosopagnosia comprises case reports and small case series. We aim to assess demographic, clinical and imaging characteristics and neurological and neuropathological disorders associated with a diagnosis of prosopagnosia in a large cohort. Patients were categorized as developmental versus acquired; those with acquired prosopagnosia were further subdivided into degenerative versus non-degenerative, based on neurological aetiology. We assessed regional involvement on [^18^F] fluorodeoxyglucose-PET and MRI of the right and left frontal, temporal, parietal and occipital lobes. The Intake and Referral Center at the Mayo Clinic identified 487 patients with possible prosopagnosia, of which 336 met study criteria for probable or definite prosopagnosia. Ten patients, 80.0% male, had developmental prosopagnosia including one with Niemann–Pick type C and another with a forkhead box G1 gene mutation. Of the 326 with acquired prosopagnosia, 235 (72.1%) were categorized as degenerative, 91 (27.9%) as non-degenerative. The most common degenerative diagnoses were posterior cortical atrophy, primary prosopagnosia syndrome, Alzheimer’s disease dementia and semantic dementia, with each diagnosis accounting for >10% of this group. The most common non-degenerative diagnoses were infarcts (ischaemic and haemorrhagic), epilepsy-related and primary brain tumours, each accounting for >10%. We identified a group of patients with non-degenerative transient prosopagnosia in which facial recognition loss improved or resolved over time. These patients had migraine-related prosopagnosia, posterior reversible encephalopathy syndrome, delirium, hypoxic encephalopathy and ischaemic infarcts. On [^18^F] fluorodeoxyglucose-PET, the temporal lobes proved to be the most frequently affected regions in 117 patients with degenerative prosopagnosia, while in 82 patients with non-degenerative prosopagnosia, MRI revealed the right temporal and right occipital lobes as most affected by a focal lesion. The most common pathological findings in those with degenerative prosopagnosia were frontotemporal lobar degeneration with hippocampal sclerosis and mixed Alzheimer’s and Lewy body disease pathology. In this large case series of patients diagnosed with prosopagnosia, we observed that facial recognition loss occurs across a wide range of acquired degenerative and non-degenerative neurological disorders, most commonly in males with developmental prosopagnosia. The right temporal and occipital lobes, and connecting fusiform gyrus, are key areas. Multiple different pathologies cause degenerative prosopagnosia.

## Teaser

Josephs and Josephs report 336 patients diagnosed with prosopagnosia over a 23-year period. Neurodegenerative diseases accounted for 70% of cases most frequently involving the temporal lobes. Non-degenerative causes accounted for 27% with structural lesions most frequently in the right temporal–occipital lobes. Developmental prosopagnosia was observed in 10 patients, mainly boys.

## Graphical Abstract

**Graphical Abstract:**
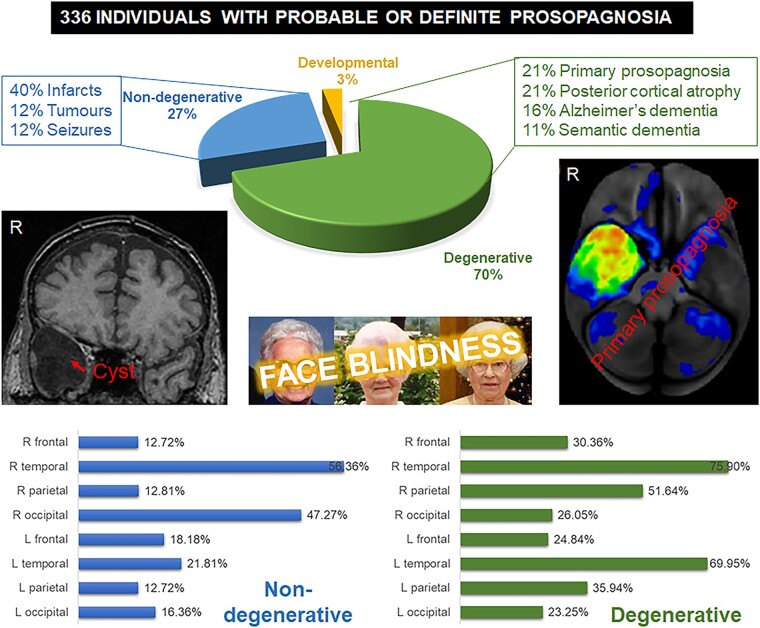

## Introduction

The term prosopagnosia, an impaired ability to recognize individuals by their face, was coined in 1947 by Bodamer,^1^ although the description of a loss of the ability to recognize human faces dates back to a case report in 1867 by ophthalmologists Quaglino and Borelli.^2^ Prosopagnosia complicates recognition of faces of previously encountered individuals, especially unfamiliar faces (i.e. faces not often seen; e.g. high school classmates or prior coworkers), and also affects recognition of familiar faces (i.e. faces encountered more often; e.g. family members). Many patients with prosopagnosia recognize their deficit. While some develop compensation strategies, such as using an individual’s voice for recognition, many patients are distressed and embarrassed by the impairment. For the first century after the recognition of prosopagnosia as a neurological symptom, the field principally focused on identifying the anatomic correlate of prosopagnosia. This period focused on the lingual and fusiform gyri of the medial occipito-temporal cortex as key regions of interest,^3,4^ but it was unclear whether bilateral or right unilateral involvement was the source. Prior to CT imaging, debate persisted about whether involvement of both hemispheres was necessary or whether just a right hemisphere focal lesion was sufficient.^3-8^ Increased use of head CT scans in neurological diseases revealed that an isolated right hemisphere lesion was sufficient to account for loss of facial recognition.^9-12^ Over the next few decades, De-Renzi and colleagues^13^ proposed that there are two different types of prosopagnosia, an apperceptive type and an associative (amnestic) type, while Warrington and colleagues^14,15^ showed that facial recognition loss differed from the loss of ability to discriminate between faces and that facial recognition can be specific to human faces. A patient with apperceptive prosopagnosia cannot identify a face due to inability to integrate the visual components (shape, colour, size, distance between parts of the face, etc.) necessary to recognize the face of a given individual. That is, the patient can provide specific details about the individual’s face if asked to describe it (e.g. a beard and a mole above the lip) but cannot identify the face when viewed. Associate prosopagnosia demonstrates loss of important information or knowledge about whose face is being perceived; the patient can match faces (hence, face perception is unaffected) but cannot provide any information about the person’s face when asked for details. Apperceptive prosopagnosia is linked to the posterior occipito-temporal region, while associative prosopagnosia is linked to the anterior temporo-occipital region.^16^ Patients can also be classified aetiologically as having developmental (congenital) prosopagnosia,^17-19^ in which difficulty with or loss of facial recognition occurs at the time of birth or in very early childhood, in the absence of a history of brain injury or identifying lesion.^20^ Developmental prosopagnosia can be of the apperceptive type or the associative type. Patients with developmental prosopagnosia may not be cognizant of their deficit or have limited insight.^21^ Some researchers propose that developmental prosopagnosia has a genetic basis.^22^

With increasing usage of head MRI scans during the late 20th century, there have been many published case reports and a few small case series of patients with brain lesions and prosopagnosia.^23^ Haemorrhagic and ischaemic strokes involving the right or bilateral posterior occipito-temporal region have been associated with prosopagnosia^24-27^ with rare left-sided involvement also reported.^28^ As a result, we now understand the importance of the posterior occipito-temporal brain region for face recognition and recognize that damage to this region results in the apperceptive type of prosopagnosia.^16^ Increasing recognition of neurodegenerative diseases^29-32^ also includes some diseases that target the anterior temporal region^33-36^ linked to the associative type of prosopagnosia. Focal brain lesions seemingly target the right occipito-temporal region, whereas neurodegenerative diseases tend to have bilateral involvement. However, the extent of this is unknown. It is also unknown whether patients with prosopagnosia from focal brain lesions have other clinical or anatomic differences compared with those with prosopagnosia from neurodegenerative diseases. Lastly, although CT and MRI scans have been utilized to study prosopagnosia, studies using more recent neuroimaging techniques are rare. One example is that of PET, which was first reported in a patient with prosopagnosia over 30 years ago.^10^

In this large case series, we aimed to address these knowledge gaps and determine the frequencies and clinical characteristics of neurological disorders associated with prosopagnosia. We also aim to compare patients with non-degenerative diseases with those with neurodegenerative diseases. We hypothesize that those with neurodegenerative diseases are older at the time of onset of the prosopagnosia compared with those with non-degenerative prosopagnosia. Another aim of this study was to assess the anatomic localization of focal, prosopagnosia-associated brain lesions on MRI scan in those with a non-neurodegenerative disease and the patterns of hypometabolism on [^18^F] fluorodeoxyglucose (FDG)-PET using advanced imaging software in those with a neurodegenerative disease. We hypothesize that those with neurodegenerative diseases will show more of a bilateral pattern of involvement compared with those with non-degenerative prosopagnosia.

## Materials and methods

### Subjects

The study was reviewed by an expedited review procedure wizard and determined to be exempt from the requirement for Institutional Review Board (IRB) approval (45 CFR 46.104d, category 4). The Intake and Referral Center at the Mayo Clinic identified all patients with possible prosopagnosia that had undergone neurologic evaluation at Mayo Clinic campuses in Rochester, MN; Scottsdale, AZ; or Jacksonville, FL, between 1 January 2000 and 31 January 2023. The system identified all patients’ medical records in which the terms ‘prosopagnosia’, ‘facial recognition’, ‘face recognition’, ‘face identification’, ‘person identification’, ‘person recognition’, ‘forgets people’, ‘doesn’t remember people’, ‘doesn’t remember face’, ‘does not remember faces’ and ‘does not remember people’ appeared. The system also searched for any patients with an ICD9 code of 368.16, psychophysical visual disturbances (ICD10:R48.3; visual agnosia) as a diagnosis. Ken.A.J. carefully reviewed medical records of all patients identified by the Intake Center to determine whether patients met criteria for probable prosopagnosia, definite prosopagnosia or no prosopagnosia. ‘Probable prosopagnosia’ was defined as a subjective complaint of facial recognition loss by the patient, caregiver or significant other, without objective evidence (i.e. no formal testing was performed to confirm prosopagnosia, or testing was performed and interpreted as within normal limits). ‘Definite prosopagnosia’ was defined as facial recognition loss as noted by the patient, caregiver or significant other with objective evidence of prosopagnosia (i.e. completed informal or formal testing for facial recognition loss and demonstrated loss of face recognition). We excluded patients whose medical records stated ‘no prosopagnosia’ or ‘no facial recognition loss’ or mentioned prosopagnosia affecting the patient’s family member instead of the patient, misinterpreted the complaint as prosopagnosia, mentioned face recognition use with a smartphone or only had difficulty with naming faces (i.e. no mentioned of loss of face recognition, ‘no prosopagnosia’).

Ken.A.J. extracted demographic and clinical data for patients meeting inclusion criteria. The Research Electronic Data Capture (REDCap) was used to capture all abstracted data for this study. These data included sex, age at onset of the prosopagnosia-associated neurological disease, age at onset of prosopagnosia, documentation of the specific face recognition-related complaint/complaints, age at the time of neurological examination, formal/informal examination of facial recognition loss and performance on testing and documentation of available imaging for review (head CT or MRI and FDG-PET). Additional clinical data extracted included the subjects’ neurological diagnosis at the time of prosopagnosia onset and the presence of other clinical characteristics including visual hallucinations, auditory delusions, ideomotor limb apraxia, other visual agnosias, homonymous haemianopsia, haemineglect, behavioural changes, personality changes, complaint-related ophthalmology evaluations and neuropathological diagnoses in all subjects who had died and completed a brain autopsy. Patients were first divided into two broad categories according to the age at onset of the loss of facial recognition.^37^ Patients with facial recognition loss at the time of birth or in early childhood were considered to have ‘developmental prosopagnosia’. Those with facial recognition loss after early childhood were categorized as ‘acquired prosopagnosia’. We then subdivided those with acquired prosopagnosia into two subcategories, degenerative and non-degenerative, based on the neurological diagnosis associated with or determined to have caused the loss of facial recognition.

### Prosopagnosia testing

Two types of facial recognition tests were administered to some patients in this cohort. In the first test type (informal), the patient was shown the face of a famous person and asked whether the patient recognizes the face. If the answer was no, the patient did not get credit. If the answer was yes, the patient was asked to provide any information they can about the person. If any information was provided supporting knowledge about the individual, credit was given; otherwise, no credit was given. There was a range of different numbers of faces (10–30 faces) and a different set of faces used in this test type over the period of study. In the second test type (formal), the patient was shown a panel of three faces in which one face was that of a famous person and asked to point to the face that is recognized. The patients were specifically told they do not need to know the name of the person, just whether they recognize any one of the three faces or not. If the patient points to the correct/famous face, credit was given; otherwise, no credit was given. Following recognition of the famous face, the patient was then asked whether they could name the person. Naming of the face was not used to determine presence of prosopagnosia but was important to exclude patients without prosopagnosia but who instead have difficulty with face naming. As previously described, this test type has a total of 10 panels of 3 faces and has been normed based on performance in 50 normal controls with ≤8 considered abnormal.^38^

### Neurological diagnosis

A board-certified neurologist (Kei.A.J.) independently reviewed the medical records of all patients included in the study to determine neurological diagnosis. This included all medical records by the patients’ neurologist, internist, psychiatrist, family medicine physician and ophthalmologist over their entire evaluation period at Mayo Clinic (range: years to decades). Acquired non-degenerative clinical diagnosis was the designation if the onset of facial recognition loss correlated with a structural lesion (e.g. ischaemic infarct, brain haemorrhage, or brain tumour) or with a neurological diagnosis not considered neurodegenerative in nature (e.g. migraine). If a patient had two non-degenerative diagnoses, e.g. a primary brain tumour and seizures, the diagnosis was linked to the one that was time-locked to the onset of facial recognition loss. For degenerative diagnoses, we applied published criteria for posterior cortical atrophy,^39^ Alzheimer’s disease dementia (also known as classic, typical or amnestic Alzheimer’s disease),^40^ semantic dementia,^41^ dementia with Lewy bodies,^42^ logopenic progressive aphasia,^43^ behavioural-variant frontotemporal dementia,^44^ Creutzfeldt–Jakob disease^45^ and corticobasal syndrome.^46^ Patients >60 years old with facial recognition loss, in the presence of an equally or more prominent relative focal loss of episodic memory (i.e. in the relative absence of any other cognitive or behavioural changes), and with little change occurring over a period of years were diagnosed with hippocampal sclerosis of aging (HSA).^47,48^ Patients with the insidious onset and worsening of isolated facial recognition loss, or in which facial recognition loss was the most prominent and profound feature, without or with minimal presence of other cognitive or behavioural features over a period of years, as originally described by Tyrrell *et al*.,^34^ were diagnosed with primary prosopagnosia syndrome (PPS).

### Neuroimaging gradings

For patients with a degenerative diagnosis as a cause for their prosopagnosia, Ken.A.J. visually reviewed individual-level patterns of hypometabolism on FDG-PET using 3D stereotactic surface projections from Cortex ID suite images whereby activity at each voxel is normalized to the pons and *Z*-scored to an age-segmented normative database (GE Healthcare, https://www.gehealthcare.co.uk/-/media/13c81ada33df479ebb5e45f450f13c1b.pdf) and graded regional hypometabolism as absent (*Z*-score: 0.0 to −2.0) versus present (*Z*-score: −3.0 to −7.0) for eight regions of interest: the left and right frontal lobes, left and right temporal lobes, left and right parietal lobes and left and right occipital lobes. For the patients with a non-degenerative diagnosis, Kei.A.J. reviewed their structural head MRI scan sequences and documented whether a structural lesion/s was present in the same eight regions of interest.

### Statistical analyses

Statistical analyses were performed using GraphPad Prism version 9.2.0 with statistical significance set at *P* < 0.05. Sex ratios and binary variables were compared between the degenerative and non-degenerative groups using the Chi-square test, while continuous variables such as age at onset were compared using the Mann–Whitney test.

## Results

### All subjects

**Figure 1:**
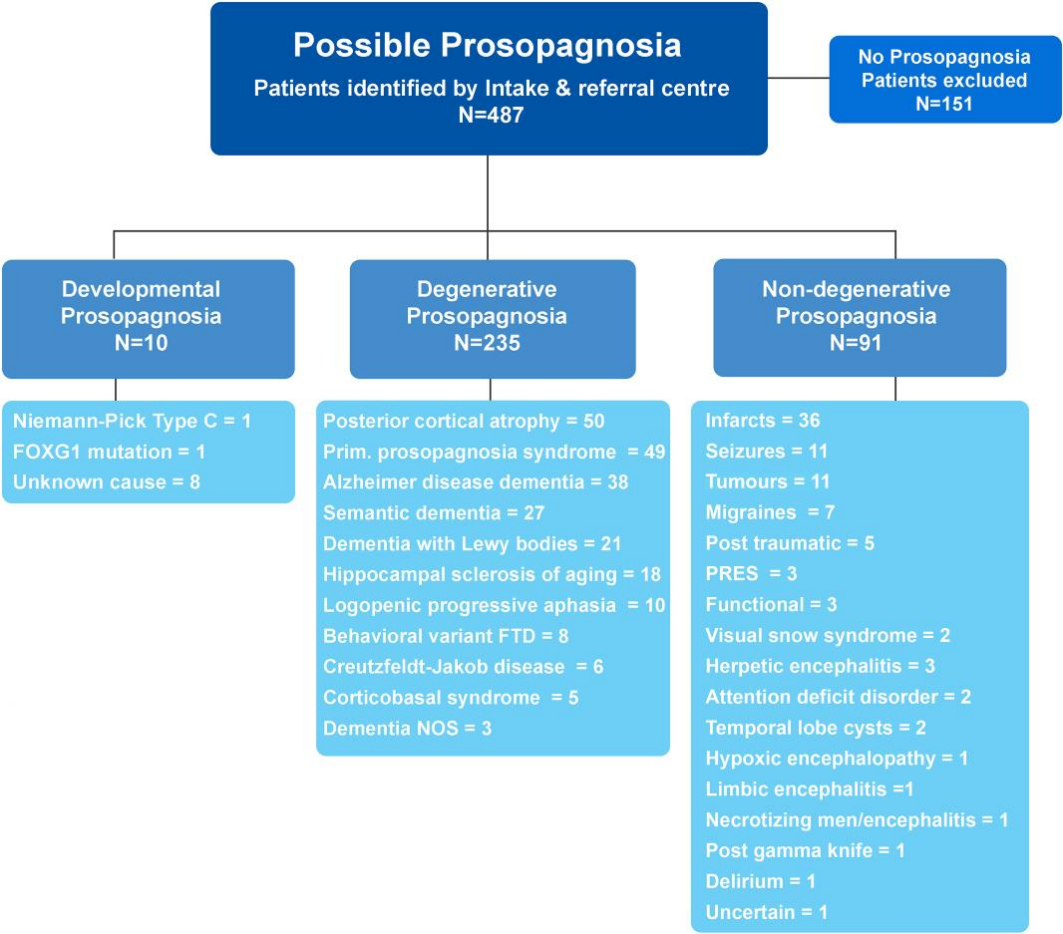
**Flow chart showing prosopagnosia diagnostic categories and neurological diagnoses within category**. Of the 333 patients with prosopagnosia included in the study, 11 had developmental prosopagnosia and 322 had an acquired prosopagnosia. Those with acquired prosopagnosia were subcategorized into acquired degenerative prosopagnosia (*n* = 233) and acquired non-degenerative prosopagnosia (*n* = 89). FOXG1, foxhead box G1; FTD, frontotemporal dementia; NOS, not otherwise specified; PRES, posterior reversible encephalopathy syndrome.

The Mayo Intake and Referral Center identified a total of 487 patients with possible prosopagnosia (Fig. 1). Of these, 151 were excluded. Ninety-two patients were excluded because their clinical notes stated ‘no prosopagnosia’, and 35 patients were excluded because they had no complaint of facial recognition loss and had completed routine objective testing for facial recognition with normal results (i.e. no evidence of prosopagnosia). Five patients were excluded because they had difficulty using face recognition technology on their smartphones. Three patients were excluded because the medical records documented prosopagnosia in the patient’s family members but not pertaining to the patient (i.e. patient’s mother, wife and patient’s grandson had prosopagnosia). Three patients were excluded because the term prosopagnosia was wrongly used (i.e. prosopagnosia was used instead of astereognosis for two patients and instead of agraphesthesia for one patient). Thirteen patients were excluded for difficulty naming faces (anomia) without objective evidence of difficulty recognizing faces (i.e. on formal testing of face recognition performance was in the normal range). The remaining 336 patients met inclusion criteria for the study. Demographic and clinical characteristics of these 336 patients are shown in Table 1. There was a higher proportion of females (55%) than males (45%). The median age at onset of prosopagnosia in the cohort was ∼66 years (age range: 0–92). Other clinical features were present in low frequencies, although a quarter of the patients had other visual agnosias, three of whom used sense of smell, or voice, for recognition. A quarter of the patients underwent formal ophthalmologic evaluation. All patients had completed at least one neuroimaging modality (CT, MRI, FDG-PET) with >90% having at least one head MRI scan available for review. Thirteen patients completed a brain autopsy at our institution with available results.

**Table 1:** Characteristics of the entire cohort with prosopagnosia

Demographic and clinical characteristics	*N* = 336
Female	184 (54.8%)
Median (range) age at onset of neurologic disorder, years	63 (0–90)
Median (range) age at onset of prosopagnosia, years	66 (0–92)
Median (range) age at time of baseline neurological examination, years	68 (7–92)
Number with acquired prosopagnosia (%)	327 (97.0%)
Number with visual hallucinations (%)	37 (11.0%)
Number with delusions (%)	14 (4.2%)
Number with ideomotor limb apraxia (%)	26 (7.7%)
Number with other types of agnosias (%)	82 (24.4%)
Number with haemianopia (%)	52 (15.5%)
Number with neglect (%)	19 (5.7%)
Number with behavioural changes (%)	69 (20.5%)
Number with personality changes (%)	77 (22.9%)
Number completing ophthalmologic examination (%)	83 (24.7%)
Number completing autopsy (%)	13 (3.9%)
Number with any imaging (CT/MRI/FDG-PET) available (%)	331 (98.5%)
Number completing head MRI available for review	309 (92.0%)
Number completing head FDG-PET available for review	127 (37.8%)

### Developmental prosopagnosia

Of the 336 patients meeting inclusion criteria, 10 were classified with developmental prosopagnosia (Fig. 1), 8 of which were male (80.0%). The median age at prosopagnosia onset in this group was 0 years (range: 0–0 years). All were thought to be born with prosopagnosia. Two of the 10 patients were tested, and both met criteria for definite prosopagnosia. One patient with developmental prosopagnosia was unaware of his deficit until he went to college. Of the 10 patients in this group, one was diagnosed with Niemann–Pick type C disease and another with a mutation in the forkhead box G1 (*FOXG1*) gene. This latter patient presented with cortical blindness, with delayed myelination observed on head MRI scan at age 2. Another patient’s father was also diagnosed with prosopagnosia.

The remaining 326 patients were categorized as having an acquired prosopagnosia. Of these 326 patients, 230 (70.6%) met criteria for definite prosopagnosia, while 96 (29.4%) met criteria for probable prosopagnosia. Of these, 182 were female (55.8%) with the median onset age of 67 (range: 7–92 years). Examples of specific complaints of facial recognition loss are described in Table 2.

**Table 2:** Examples of specific complaints of face blindness and associated diagnoses

Face blindness description by patient or spouse	Associated diagnosis
He does not recognize people in his hometown whom he has not seen for a while and shops in other towns to avoid running into people he may know.	Attention deficit disorder
Difficulty recognizing familiar people including extended family members and the families of her students.	Epilepsy with focal partial seizures
When she looked at some family members or TV characters, they sometimes would appear like ‘cubism’.	Medically intractable epilepsy
Needs to use his sense of smell and individuals voice to recognize people.	Post-herpetic encephalitis
Initially after stroke, had difficulty with facial recognition, but it has improved.	Right posterior cerebral artery ischaemic infarct
He did not recognize his own image in the mirror.	Right posterior cerebral artery ischaemic infarct
Could not recognize my wife’s face in the grocery store.	Traumatic brain injury
He coached a rowing team and had a spell where he was unable to recognize his team.	Visual migraines
Husband says she has trouble recognizing people she knows until she is reminded of their name.	Alzheimer’s disease dementia
Fails to recognize her husband and has called the police thinking he was an intruder.	Alzheimer’s disease dementia
Intermittent difficult recognizing people especially when waking from sleep.	Alzheimer’s disease dementia
Difficult recognizing family members, especially her grandchildren.	Corticobasal syndrome
Did not recognize the face of her daughter.	Dementia with Lewy bodies
Trouble recognizing his wife at certain times.	Hippocampal sclerosis of aging
Did not recognize his granddaughter and son-in-law when they visited unannounced.	Hippocampal sclerosis of aging
Difficulty recognizing grandkids and people he frequently visits.	Logopenic progressive aphasia
Difficulty recognizing his daughters and only recognizes them by their voices.	Posterior cortical atrophy
‘She has to really look at you to recognize you’, and she may have a conversation with someone and then later ask ‘Who was that?’	Posterior cortical atrophy
‘Not easy for her to recognize her children without smelling them’.	Posterior cortical atrophy
Sudden onset of trouble recognizing faces that lasted for a day	Posterior reversible encephalopathy syndrome
Asked her daughter where her daughter lives, but she only has one daughter.	Primary prosopagnosia syndrome
Cannot recognize familiar faces such as family members in photographs.	Primary prosopagnosia syndrome
Unable to recognize the students that she was teaching.	Primary prosopagnosia syndrome
His wife says he has difficulty recognizing familiar people who are encountered out of context.	Semantic dementia
He did not recognize the neurologist that he has seen many times before.	Semantic dementia
Cannot pick his family out from a crowd or recognize anyone in family pictures.	Developmental prosopagnosia

### Prosopagnosia due to neurodegenerative disease

Of the 326 patients with acquired prosopagnosia, 235 had a degenerative diagnosis. The three most common degenerative diagnoses were posterior cortical atrophy, PPS and Alzheimer’s disease dementia, with each diagnosis accounting for >10% of the neurodegenerative aetiologies. Other, less common, diagnoses are shown in Fig. 1. Demographic and clinical characteristics across all the degenerative diagnoses are shown in Supplementary Table 1. Patients with HSA had the oldest median age of prosopagnosia onset (82 years), which was 20 years older than those with a diagnosis of semantic dementia, which had the youngest age of onset (62 years). Visual hallucinations were most common in patients with dementia with Lewy bodies (71.4%), limb apraxia was most common in those with corticobasal syndrome (100%), other visual agnosias were most common in those with posterior cortical atrophy (73.5%), and behavioural and personality changes were most common in behavioural-variant frontotemporal dementia (100% for both). In patients with degenerative prosopagnosia, the prosopagnosia was stable or would worsen over time. Eight patients may have had Capgras syndrome, which mimics prosopagnosia. Of these eight patients, six were diagnosed with dementia with Lewy bodies, one with posterior cortical atrophy and one with Alzheimer’s disease dementia.

### Prosopagnosia due to non-degenerative disease

Ninety-one patients had a non-degenerative diagnosis (Fig. 1). The most common non-degenerative diagnoses were infarcts (ischaemic and haemorrhagic infarcts) followed by epilepsy/seizure-related diseases and primary brain tumours. Two patients were diagnosed with mitochondrial encephalopathy with lactic acidosis and strokes, one presenting with infarcts and the other with seizures. Other non-degenerative diagnoses, including migraine-related prosopagnosia, were less common. (See Supplementary Table 2 for medical summaries of presenting features.) Two patients with migraine-associated prosopagnosia were diagnosed with stroke-like migraine attacks after radiation therapy (SMART) syndrome. Five patients developed prosopagnosia immediately after a traumatic brain injury. Three patients with non-degenerative prosopagnosia were diagnosed with somatization/functional disorder; all were female with ages of onset of 42 years, 54 years and 60 years old. In all three patients, the onset of the prosopagnosia was described as acute, with one patient (age 42) describing onset immediately post-partum. Three patients were diagnosed with posterior reversible encephalopathy syndrome (PRES), one in which Capgras was considered mimicking prosopagnosia and three with post-herpetic encephalitis. Two patients each had visual snow syndrome and attention deficit disorder. The patients with visual snow syndrome described their vision like ‘snow on a black and white television’, with one patient also stating, ‘sometimes it’s like looking through bubble wrap’. Both of these patients also had difficulty with visual motion, and both had ophthalmologic consultations confirming the diagnosis.

Some patients with infarcts reported that the prosopagnosia improved (but did not always resolve) over time. Patients with migraine-associated prosopagnosia, including one with SMART syndrome, had transient prosopagnosia that completely resolved after several hours; although in many instances, prosopagnosia did recur. Similarly, the three patients with PRES, one with delirium (due to hypernatremia and drug overdose), one with an infarct and one with hypoxic encephalopathy post-ventricular fibrillation arrest, reported transient prosopagnosia with subsequent complete resolution occurring over a period of days to weeks.

### Comparing degenerative and non-degenerative diseases

Demographic and clinical characteristics comparing the degenerative group and the non-degenerative group are shown in Table 3. Definite prosopagnosia was significantly more frequent in the group of degenerative diagnoses than the non-degenerative diagnoses (*P* < 0.0001). The median age at onset of the neurological diagnosis, median age at onset of prosopagnosia and median age at the time of neurological examination were all older in the degenerative group (*P* < 0.0001). Nineteen patients with a degenerative diagnosis (8.1%) had homonymous haemianopsia, whereas 34 patients with a non-degenerative diagnosis (37.4) had homonymous haemianopsia; 21 of those 34 had haemorrhage or ischaemic infarcts (*P* < 0.0001). Behaviour and personality changes were more commonly seen in the degenerative group (*P* < 0.0001), while the number of patients who underwent ophthalmologic examination was more common in the non-degenerative group (*P* = 0.04).

**Table 3:** Characteristics of those with acquired prosopagnosia with and without a degenerative diagnosis

Characteristics	Degenerative (*N* = 235)	Non-degenerative (*N* = 91)	*P*-value
Probable: definite prosopagnosia	24:211	72:19	<0.0001
Female sex	133 (56.6%)	49 (53.8%)	0.6161
Median age at disease onset (range), years	65 (24–90)	61 (9–89)	<0.0001
Median age at onset of prosopagnosia, years	69 (39–92)	61 (12–89)	<0.0001
Median age at neurological examination, years	70.5 (26–92)	62 (12–89)	<0.0001
Number with visual hallucinations (%)	28 (11.9%)	9 (9.9%)	0.7007
Number with delusions (%)	12 (5.1%)	2 (2.2%)	0.2559
Number with ideomotor limb apraxia (%)	24 (10.2%)	4 (4.4%)	0.0982
Number with visual agnosia (%)^a^	49 (20.9%)	20 (22.0%)	0.5053
Number with homonomous haemianopia (%)	19 (8.0%)	34 (37.3%)	<0.0001
Number with neglect (%)	12 (5.1%)	6 (6.6%)	0.5783
Number with behavioural changes (%)	66 (28.1%)	4 (4.4%)	<0.0001
Number with personality changes (%)	73 (31.1%)	5 (5.5%)	<0.0001
Number completing ophthalmologic examinations (%)	49 (20.9%)	30 (33.0%)	0.0181
Number completing autopsy (%)	13 (5.5%)	3 (3.3%)	0.1570

^a^Visual agnosia = apperceptive, simultagnosia or motion agnosia.

**Figure 2:**
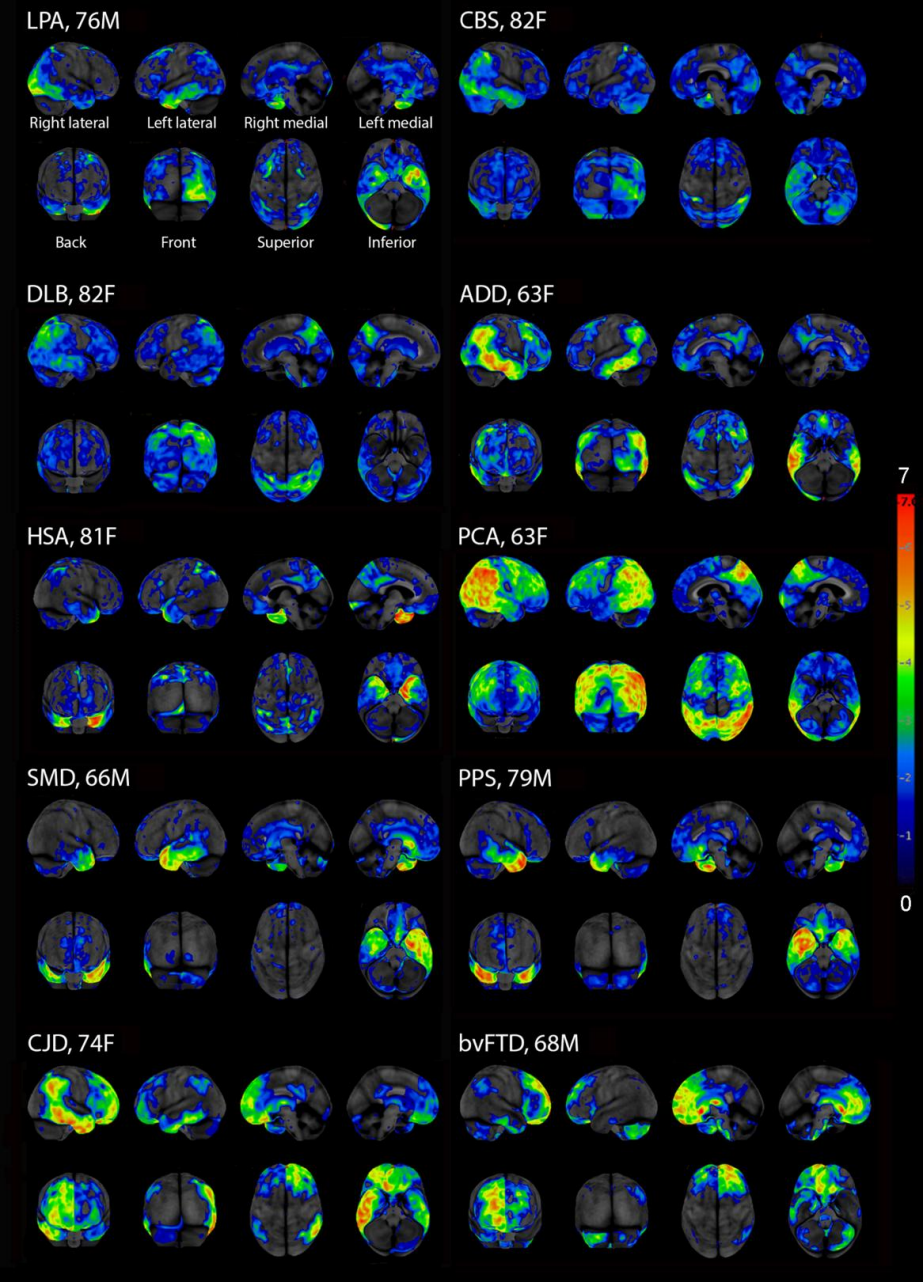
**FDG-PET of hypometabolic areas in patients with neurodegenerative diagnoses**. This figure demonstrates regional hypometabolism in patients with neurodegenerative prosopagnosia. Shown for each image are the patient’s sex, age at onset of prosopagnosia and the neurological diagnosis. Colours represent severity/degree of hypometabolism based on *Z*-score difference in metabolism between the patient’s uptake and mean uptake of a group of age-matched health controls. All patients show some degree of cortical hypometabolism but with different patterns of regional involvement. ADD, Alzheimer’s disease dementia; bvFTD, behavioural-variant frontotemporal dementia; CBS, corticobasal syndrome; CJD, Creutzfeldt–Jakob disease; DLB, dementia with Lewy bodies; HSA, hippocampal sclerosis of aging; LPA, logopenic progressive aphasia; PCA, posterior cortical atrophy; PPS, Progressive apraxia syndrome; SMD, semantic dementia.

**Figure 3:**
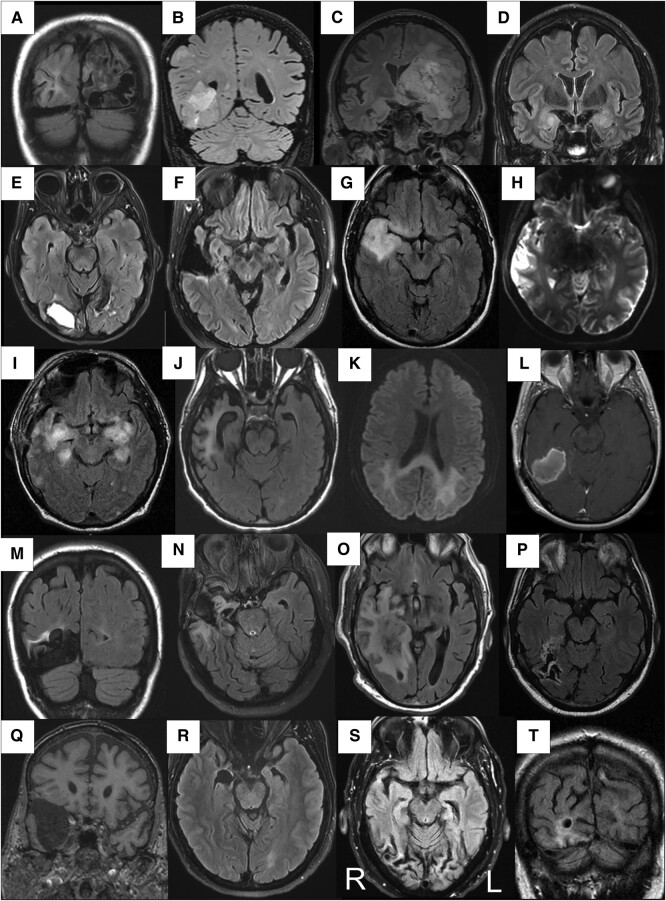
**MRI scans of structural abnormalities in patients with a non-degenerative diagnosis**. This figure shows a wide range of diverse types of lesions identified on head MRI scan in the patients with non-degenerative prosopagnosia. R, right hemisphere; L, left hemisphere. All images are fluid-attenuated inversion recovery (FLAIR) sequences except for images **H** and **K** (diffusion-weighted images), **L** (T1 + gadolinium) and **Q** (3D fobl). Epilepsy-related lesions associated with prosopagnosia are shown in panels **A**, **F** and **M** (area of encephalomalacia). Tumour-related lesions associated with prosopagnosia are shown in panels **B** and **C** (glioblastoma multiforme), **G** and **L** (oligodendroglioma) and **O**. Other lesion-related prosopagnosias are shown in **D** (limbic encephalitis), **E** (stroke), **H** (mitochondrial encephalopathy, lactic acidosis and stroke-like episodes), **I** (necrotizing meningoencephalitis), **J** (post-herpetic encephalomalacia), **K** (toxic encephalopathy), **N** (right temporal lesion), **O** (mass of undermined aetiology), **P** (post-stroke encephalomalacia), **Q** (temporal lobe cyst), **R** and **S** (post-traumatic encephalomalacia) and **T** (post-traumatic contusion). All panels show lesions affecting either the right temporal lobe or the right occipital lobe except for **C** that shows an extensive infiltrative glioblastoma multiforme located primarily in the left hemisphere. All images shown are from adult except for **A**, which is from an adult patient with developmental prosopagnosia, whose MRI scan at age 7 is shown.

Patients with a degenerative diagnosis accounting for their prosopagnosia who completed FDG-PET scans numbered 117. Representative 3D stereotactic-surfaces projections from Cortex ID suite for the different degenerative diagnoses are shown in Fig. 2. Most patients with a non-degenerative diagnosis (*n* = 81) had a head MRI available for review; the remainder had MRIs reports without images to review. Representative structural abnormalities in the patients with non-degenerative diagnoses are shown in Fig. 3. Of the 91 patients with a non-degenerative prosopagnosia who had completed structural head imaging (MRI or CT scan) showing a lesion (*n* = 66), all but 5 patients (7.7%) had a structural lesion that involved the right temporal or right occipital lobe.

### Ophthalmologic findings

Eighty-three patients, 4 with developmental prosopagnosia and 79 with acquired prosopagnosia (30 non-degenerative and 49 degenerative), underwent an ophthalmologic evaluation at our institution. One of the four with development prosopagnosia had optic atrophy. The most common findings in those with acquired prosopagnosia were cataracts in 28 (35.4%) patients, glaucoma in 11 (13.9%) and macular degeneration in 6 (7.6%). Ten patients had other ocular disorders including two each with scleritis, retinal detachment and vitreous detachment and one each with chorioretinopathy, Fuchs’ endothelial dystrophy, subconjunctival haemorrhage and melanoma-associated retinopathy.

### Pathological findings

**Figure 4:**
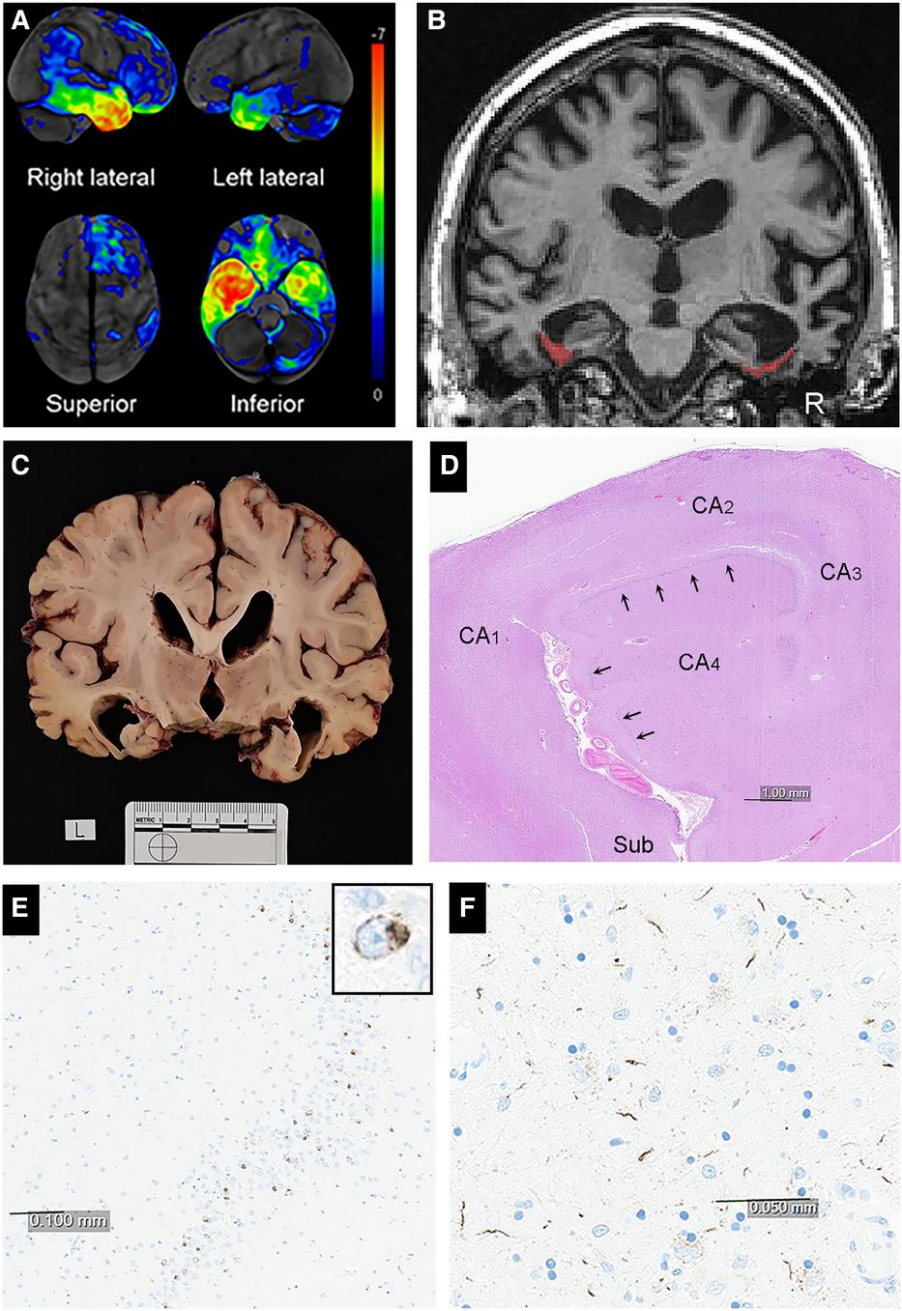
**FDG-PET, head MRI, gross and histological findings in a patient with PPS**. There is marked to severe hypometabolism of the anterior medial temporal lobes, right > left on FDG-PET (**A**). MRI (**B**) and gross pathological images (**C**) reveal atrophy of anterior medial temporal lobe regions including severe atrophy of the right fusiform gyrus. Histological findings include the presence of neuronal loss and gliosis in the CA1 and subicular regions of the hippocampus (**D**) as well as TDP-43 immunoreactive small rounded or grain-like inclusions (brown spots) in the dentate gyrus of the right hippocampus (**E**) and long thin dystrophic neurites (brown thread-like squiggles) in the right temporal neocortex (**F**) consistent with a pathological diagnosis of FTLD-TDP type C. On MRI, the fusiform gyri are highlighted in red. Black arrows in **E** point to the dentate granule cells of the hippocampus. TDP-43 immunohistochemistry, with a haematoxylin counter stain, was perform using phosphorylated TDP-43 (pS409/410, mouse monoclonal, 1:5000, Cosmo Bio, Tokyo, Japan).

Thirteen patients with degenerative prosopagnosia had a brain autopsy at our institution (Supplementary Table 3). The most common histopathological finding was frontotemporal lobar degeneration (FTLD) with TAR DNA binding protein 43 (TDP-43) inclusions (*n* = 8) with hippocampal sclerosis (*n* = 5/8), FTLD-tau (*n* = 4), mixed high likelihood Alzheimer’s disease and Lewy body disease (*n* = 4). Three of the four patients with PPS had FTLD-TDP-43 type C pathology. Other pathologies included diffuse argyrophilic grain disease, globular glial tauopathy and Pick’s disease. In all cases of FTLD pathology, there was marked to severe atrophy of anterior medial temporal lobe regions, including the fusiform gyrus (Fig. 4).

### Neuroimaging gradings

**Figure 5:**
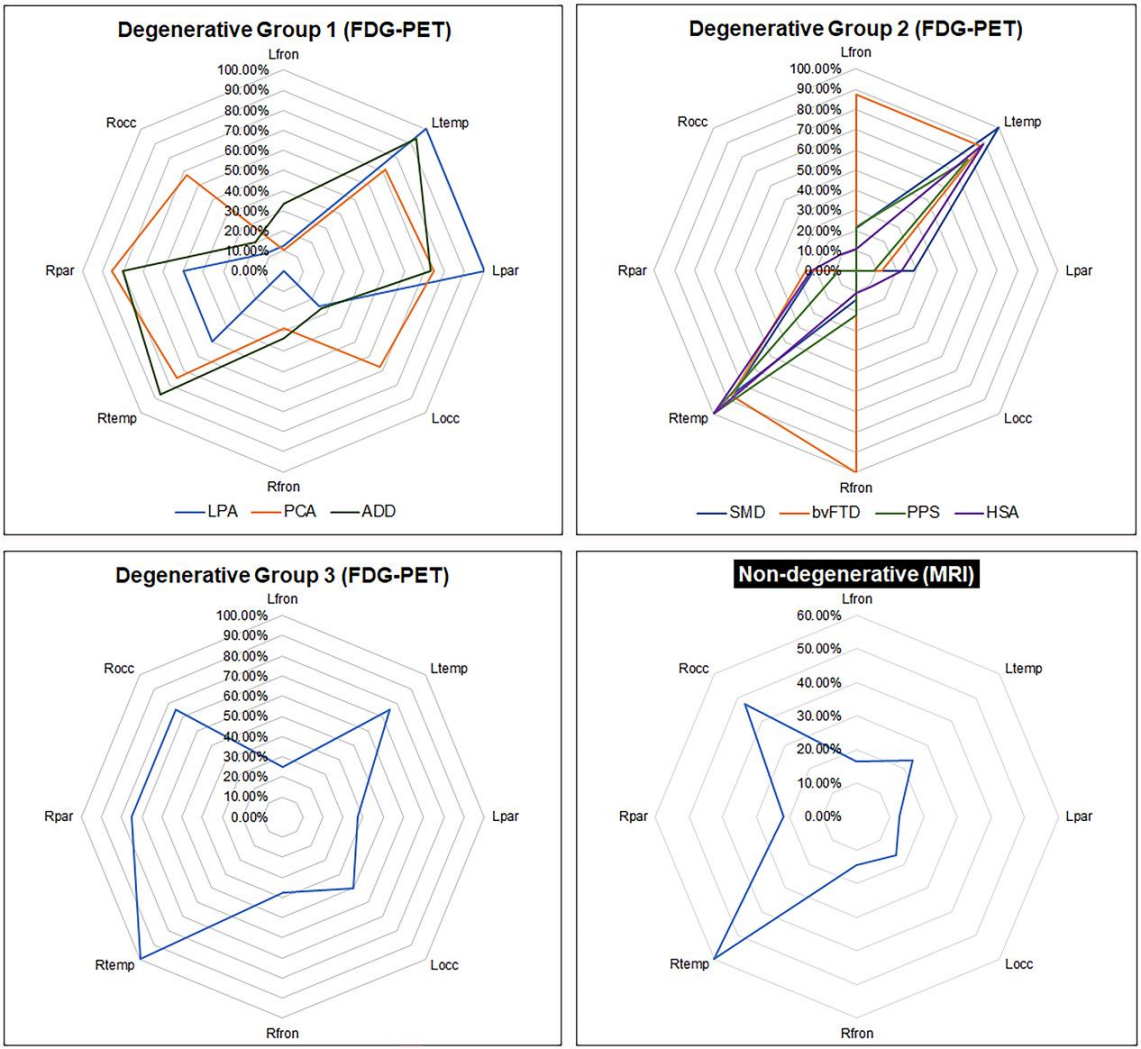
**Radar plots showing regional hypometabolism and lesion location**. This figure shows the proportion of patients that showed involvement of eight brain regions: left and right frontal, temporal, parietal and occipital lobes. For the 117 patients with a neurodegenerative disease, the plots show the proportion of patients with moderate-to-severe hypometabolism on FDG-PET in each brain region. The neurodegenerative diseases are split into three groups: Group 1, ‘the Alzheimer’s disease group’, includes logopenic progressive aphasia (LPA), posterior cortical atrophy (PCA) and Alzheimer’s disease dementia (ADD). Group 2, ‘the frontotemporal lobar degeneration group’, includes semantic dementia (SMD), behavioural-variant frontotemporal dementia (bvFTD), primary prosopagnosia syndrome (PPS) and hippocampal sclerosis of aging (HSA). Group 3, ‘the mixed group’, includes dementia with Lewy bodies (DLB), corticobasal syndrome (CBS), Creutzfeldt–Jakob disease (CJD) and dementia not otherwise specified (DEM NOS). For example, in Degenerative group 1, the right parietal lobe showed hypometabolism in >80% of the PCA patients (orange web), while 100% of the LPA patients (blue web) showed hypometabolism of the left temporal and parietal lobes. For the 81 patients with a non-degenerative disease, the plot shows the proportion with a focal lesion on MRI affecting each of the eight brain regions. Lesions were most frequently observed in the right temporal and occipital lobes, with ∼60% of patients having a lesion that affected the right temporal lobe and ∼50% having a lesion that affected the right occipital lobe. Note that a lesion/s could affect multiple regions. For example, a large lesion (e.g. a tumour) could involve both right temporal and right occipital regions and hence would contribute to the frequency of both regions for that one patient.

FDG-PET results for the 117 patients with degenerative diagnoses are reported in 3 groups (Fig. 5). Group 1 consisted of three diagnoses considered to be on the Alzheimer’s disease spectrum, Group 2 consisted of four diagnoses considered to be on the frontotemporal dementia spectrum, and Group 3 consisted of the remaining diagnoses. Group 1 diagnoses showed greatest hypometabolism in bilateral temporal lobes followed by involvement of bilateral occipital and parietal lobes. The logopenic progressive aphasia group showed less right-sided involvement compared with the other two groups. Group 2 diagnoses showed predominantly bilateral temporal lobe hypometabolism. The frontal lobes were involved only in patients diagnosed with behavioural-variant frontotemporal dementia. Group 3 findings were limited by small sample sizes but did show involvement mainly on the right especially right temporal, followed by right parietal and right occipital.

MRI results for the 81 patients with a non-degenerative diagnosis are reported as 1 group (Fig. 5). We found a striking asymmetry of involvement of the right temporal lobe followed by involvement of the right occipital lobe. Lesions were most common in the right occipital lobe (68%) in those with infarcts and in the right temporal lobe in those with tumours (50%) and epilepsy (75%).

## Discussion

In this large case series of 336 patients diagnosed with prosopagnosia, we found many different neurological causes that mostly occurred later in life although we also identified patients with prosopagnosia occurring from the time of birth. We observed some patients diagnosed with prosopagnosia using alternative means, such as smell or voice, for person recognition. We identified a wide range of degenerative and non-degenerative aetiologies, usually affecting the right anterior medial temporal and/or right occipital lobes. We observed that prosopagnosia is not always a permanent symptom but, when associated with certain non-degenerative aetiologies, may improve or resolve with time. We discovered that different underlying pathologies are associated with degenerative prosopagnosia, the most common being FTLD.

Across the entire cohort of patients diagnosed with prosopagnosia, there was a slightly higher frequency of women. A higher frequency of women was also observed in those with degenerative and non-degenerative acquired prosopagnosia but not in developmental prosopagnosia, which was more frequent in boys as has been previously reported.^37^ Two patients with developmental prosopagnosia were found to harbour a genetic mutation, one in the Niemann–Pick type C gene^49,50^ and the other in the *FOXG1* gene.^51^ Niemann–Pick type C is a lysosomal storage disease often affecting children in early childhood, where presenting signs of dystonia, ataxia, seizures, eye movement abnormalities, developmental delay and hepatosplenomegaly are diagnostic clues.^52^ Patients with mutations in the *FOXG1* gene typically present with microcephaly, aphasia, movement disorders, epilepsy and developmental delay and an associated cerebral malformation.^53^ Neither of these two genetic diseases has been previously linked to prosopagnosia, but given our findings, both should be considered in the evaluation of developmental prosopagnosia. Of note, a third patient in our cohort with developmental prosopagnosia had a father with prosopagnosia, which suggests an underlying genetic cause. Hence, we strongly support genetic counselling and screening for all patients with developmental prosopagnosia. Developmental prosopagnosia has been characterized as lacking obvious lesions on brain imaging.^19,20^ However, one of our patients with *FOXG1* mutation had abnormalities on MRI. This suggests that brain imaging although often normal may not always be normal in patients with developmental prosopagnosia.

As a category, degenerative disease was the most common diagnosis. Within this category, prosopagnosia is often an accompanying symptom that is present in addition to a constellation of other signs and symptoms that are core features of the syndrome. In behavioural-variant frontotemporal dementia, for example, all patients had behavioural and personality change, and similarly, all diagnosed with corticobasal syndrome had limb apraxia. Prosopagnosia has been reported in a handful of patients with posterior cortical atrophy^54,55^ with the apperceptive type considered a feature of the syndrome.^39^ The associative type of prosopagnosia is considered a core feature of semantic dementia,^20,41^ especially when the right anterior temporal lobe is affected.^33,56^ Recent studies have identified prosopagnosia as a key feature of the right temporal variant of frontotemporal dementia,^35^ which arguably is an anatomic variant of semantic dementia.^57,58^ The other syndromic neurodegenerative diagnoses encountered in our study have less commonly, or have not been previously, reported to correlate with prosopagnosia including Alzheimer’s disease dementia,^30,59,60^ dementia with Lewy bodies,^61^ corticobasal syndrome, behavioural-variant frontotemporal dementia,^62^ logopenic progressive aphasia and Creutzfeldt–Jakob disease.^63^ This may be because these neurodegenerative diseases tend to target the left hemisphere, frontal lobes or dorsal stream of the occipito-parietal cortex. Hence, the ventral right occipito-temporal cortex is typically spared in these degenerative diseases.

Unlike the other neurodegenerative disorders in this cohort, PPS and HSA target the anterior medial temporal lobes including the fusiform gyrus. In PPS and HSA, there are few additional symptoms present. In PPS, prosopagnosia is the primary and most prominent complaint and, in some instances, the only complaint and finding. Similar patients have been described in the literature, although other diagnostic labels have been assigned including progressive prosopagnosia^34,64,65^ and primary progressive prosopagnosia.^29^ In HSA, unlike in PPS, episodic memory loss tends to be the most prominent presenting feature and may be present for years prior to the onset of the prosopagnosia. In general, patients with HSA tend to be older than those with PPS at onset.

The diagnosis of prosopagnosia in neurodegenerative disease is somewhat controversial as it is not universally accepted that patients with difficulty perceiving faces in the presence of other visual and cognitive dysfunction that could affect face perception should be diagnosed as having apperceptive prosopagnosia.^66^ This is particularly important when considering making a diagnosis of apperceptive prosopagnosia in patients with posterior cortical atrophy and other Alzheimer’s disease variants where simultagnosia, visuospatial and visuoperceptual deficits are present. We caution against making a diagnosis of apperceptive prosopagnosia unless it can be demonstrated that the severity of the loss of face recognition far outweighs the severity of all other visual dysfunction and the severity of all other visual dysfunction being no more than mild. It should also be a point of discussion whether it is appropriate to render a diagnosis of associative prosopagnosia when other cognitive deficits, such as impairment of episodic memory, are present and more severe. Another point of recent discussion pertains to diagnosing associative prosopagnosia in patients with right temporal frontotemporal dementia and semantic dementia. Some investigators have argued that such a diagnosis is misleading given that loss of face recognition occurs in the context of a general loss of knowledge about the person, and hence, it has been proposed that a more appropriate term would be loss of person-specific knowledge.^67^ Whether this general loss of knowledge occurs in all patients with PPS is unclear.

Another interesting observation in our degenerative cohort was difficulty differentiating prosopagnosia from Capgras syndrome, especially in patients diagnosed with dementia with Lewy bodies. Capgras syndrome is a delusional misidentification symptom in which there is a belief that an individual has been replaced by an imposter.^68^ It strongly correlates with dementia with Lewy disease,^69^ and hence, differentiating Capgras syndrome from prosopagnosia in patients with dementia with Lewy bodies can be difficult. Furthermore, both symptoms may be present as has been shown with Capgras syndrome patients having deficits in face recognition.^61^ In our patients with dementia with Lewy bodies, some underwent formal testing for prosopagnosia with diagnosed loss of facial recognition and, hence, met our criteria for definite prosopagnosia. Given the well-known association between Capgras syndrome and dementia with Lewy bodies, we caution diagnosing prosopagnosia and encourage formal facial recognition testings to determine whether one or both symptoms are present.

Non-degenerative causes for prosopagnosia were common, accounting for over a quarter of diagnoses in our cohort. The most common lesion in the cohort with non-degenerative prosopagnosia was infarcts followed by seizure/epilepsy-related prosopagnosia and prosopagnosia secondary to brain tumours. Prosopagnosia as a presentation of a seizure disorder or as a post-ictal phenomenon has been reported.^70-72^ Similar to infarcts,^23-27^ published case reports have linked brain tumours with prosopagnosia^73^ as well as prosopagnosia arising after resection of a primary brain tumour^74,75^ or resection for seizure treatment. One of our patients developed prosopagnosia after gamma knife surgery for resection of an arteriovenous malformation in the right basal ganglia suggesting that surgical procedures targeting lesions in the right temporal–occipital area can cause collateral damage resulting in prosopagnosia. This finding has important implications for preoperative planning for excision of lesions affecting the right anterior temporal lobe, the right occipital lobe, lesions close to the fusiform gyrus and even those in deep subcortical structures in the right hemisphere. From our study, it appears that seizure-related prosopagnosia and tumours were most often associated with involvement of the right anterior medial temporal lobe including the fusiform gyrus, while infarcts were most commonly located in the right occipital lobe.

There were many other, less common diagnostic associations with prosopagnosia including SMART syndrome, mitochondrial encephalitis with lactic acidosis and stroke-like episodes, limbic encephalitis, post-herpetic encephalitis, post-hypoxic encephalopathy and post-traumatic brain injury. There have been two reported cases of limbic encephalitis presenting with prosopagnosia, one due to anti-adenylate kinase antibodies^76^ and the other due to mGluR5 antibodies.^77^ There has also been one report of mitochondrial encephalitis lactic acidosis and strokes-like episodes,^78^ one with post-herpetic encephalitis^79^ and one of post-traumatic prosopagnia.^80^ We also encountered novel associations such as patients with attention deficit disorder and patients with non-organic neurological diagnoses. These diagnoses are not expected to be associated with prosopagnosia, although there have been reports of an association between prosopagnosia and schizophrenia^81,82^ due to structural and functional deficits of the fusiform gyrus.^83,84^

Two of the most interesting findings in the patients with a non-degenerative diagnosis were the association of prosopagnosia with migraines and the observation that prosopagnosia can be transient and resolve over time. Prosopagnosia as a feature of classic migraine is rarely described in the literature^85-87^ since the first report over two decades ago.^88^ We did encounter patients with classic migraines in which the prosopagnosia was a feature of aura. We also identified two patients who developed prosopagnosia in the context of migraines due to SMART syndrome. Both patients had received radiation treatment after resection for a cerebellar astrocytoma, which is one of the key features for the diagnosis of SMART syndrome.^89^ It is unknown how common patients with SMART syndrome develop prosopagnosia, but given our findings, we recommend that patients diagnosed with SMART syndrome be formally tested for prosopagnosia.

We were surprised to encounter as many patients as we did with transient prosopagnosia or prosopagnosia that improved over time. Transient prosopagnosia has been rarely reported in the literature in single case reports, where it has been found to occur in the setting of Parkinson’s disease,^31^ normal pressure hydrocephalus^90^ and after ischaemic stroke.^91^ Stimulation of the right anterior fusiform gyrus can also lead to transient prosopagnosia.^92,93^ A fair number of our patients reported improvement or resolution of their prosopagnosia over time, sometimes after receiving treatment for their primary diagnosis such as in a patient with SMART syndrome who received steroids. Predicting transient prosopagnosia is clinically useful for prognostication. Potential improvement of prosopagnosia should therefore be discussed with patients who have diagnoses associated with improvement or resolution of prosopagnosia such as infarcts, migraines, seizures, SMART syndrome, delirium and PRES.

Two neuroimaging widely available tests in clinical practice are MRI head scan and FDG-PET scan. Results from both neuroimaging analyses show that prosopagnosia strongly correlates with involvement of the anterior medial temporal and occipital lobes. In degenerative diseases, we found involvement in these regions in both hemispheres, which is not surprising given that these are degenerative diseases whereby progression from onset to time of evaluation would be expected to affect other brain regions. We also found regional patterns of hypometabolism on FDG-PET supporting the clinical diagnoses of posterior cortical atrophy, semantic dementia and behavioural-variant frontotemporal dementia. Interestingly, patients with PPS showed moderate-marked, primarily right or right-greater-than-left anterior medial temporal lobe hypometabolism, which differed from those with HSA, where we found left-greater-than-right temporal lobe hypometabolism. Unlike with FDG-PET, regional involvement was usually observed only in the right hemisphere on MRI in non-degenerative cases consistent with our hypothesis. Only in rare circumstances, especially in patients with glioblastoma multiforme with mass effect or midline shift, was there a left hemisphere focus. Our neuroimaging results are, therefore, highly consistent with the existing literature supporting the occipital lobe and the anterior temporal lobe including the fusiform gyrus as the anatomic correlate.

We reviewed the pathological findings in a small subset of patients who died and had an antemortem neurodegenerative diagnosis. The most common pathological diagnosis was FTLD associated with the protein TDP-43 (type A and type C)^94^ and FTLD associated with the microtubule-associated protein, tau^95^ (diffuse argyrophilic grain disease^96^ and globular glial tauopathy^97^) targeting the anterior medial temporal lobes including the fusiform gyri.^98,99^ We also encountered a high proportion of patients with hippocampal sclerosis co-pathology.^47^ To our knowledge, there is no degenerative pathology that selectively targets the fusiform gyrus in isolation over the entire disease course. However, it is likely that neurodegeneration preferentially targets the fusiform gyrus early in the degenerative process, in some patients with prosopagnosia. We did not find hippocampal sclerosis or Alzheimer’s disease in isolation, suggesting that such isolated pathologies may not be, or rarely are, associated with prosopagnosia.

Almost a quarter of the patients in our cohort sought ophthalmologic evaluation for their deficit. The most common ophthalmologic findings were cataracts and glaucoma, which have not been reported to be associated with a facial recognition loss, except when the cataracts are congenital.^100^ One patient with developmental prosopagnosia had optic atrophy, and six patients with acquired prosopagnosia had macular degeneration, which has been linked with reduced facial recognition.^101^ Ophthalmologic examinations can document ocular pathologies and visual field deficits. We observed that a homonymous haemianopsia occurred more frequently in our patients with a non-degenerative disease (especially infarcts) compared with those with a neurodegenerative disease (including posterior cortical atrophy).

Given the large size of the cohort, which is a strength of our study, the findings are generalizable to patients with prosopagnosia and all types of neurological disease. Another strength is the substantial number who completed brain imaging. The retrospective design of the study is a limitation. Not all patients completed a standardized objective measure of prosopagnosia, and some tests lack known sensitivity and specificity. Furthermore, tests changed over the 23 years of the study which prevented us from harmonizing them and providing information on prosopagnosia severity. Another limitation of the study was the absence of testing to determine whether those with definitive prosopagnosia had an apperceptive or associative prosopagnosia. Given the small sample size of patients who underwent pathological examination, we cannot exclude the possibility that the diagnoses may not generalize to other cohorts.

## Conclusion

Prosopagnosia can be developmental from birth or acquired later in life across a vast range of neurological disorders including neurodegenerative diseases from different underlying neuropathologies, focal brain lesions, non-degenerative non-lesion disorders such as epilepsy and migraine and even functional disorders. It can also be transient or may improve over time. Involvement of the right anterior medial temporal and occipital lobes is the key regions accounting for this symptom.

## Supplementary Material

fcae002_Supplementary_Data

## Data Availability

The data supporting the findings of this study are available from the corresponding author, upon reasonable request.
