# Supplementary material for: Prosopagnosia: face blindness and its association with neurological disorders
Source: Brain Commun. 2024 Jan 5;6(1):fcae002. doi: 10.1093/braincomms/fcae002 (PMC10901275; doi:10.1093/braincomms/fcae002)
Supplement: fcae002_Supplementary_Data [file fcae002_supplementary_data.docx]

**Supplementary Material**

**Content**

**Supplementary Table 1: Demographics & characteristics by neurodegenerative disease**

**Supplementary Table 2: Clinical summaries of migraine associated prosopagnosia**

**Supplementary Table 3: Pathological findings in 13 patients with prosopagnosia**

**Supplementary Table 1: Demographics and characteristics by neurodegenerative diagnosis**

|  | PCA  N=50 | PPS  N=49 | ADD  N= 38 | SMD  N=27 | DLB  N=21 | HSA  N=18 | LPA  N=10 | bvFTD  N=8 | CJD  N=6 | CBS  N=5 | DEM NOS  N=3 |
| --- | --- | --- | --- | --- | --- | --- | --- | --- | --- | --- | --- |
| Female | 38  (76.0%) | 29  (59.1%) | 22  (57.9%) | 11  (40.7%) | 9  (42.9%) | 10  (55.6%) | 5  (50.0%) | 3  (37.5%) | 3  (50.0%) | 3  (60.0%) | 0  (0.0%) |
| Median age at disease onset (Range) years | 60  (38-77) | 64  (47-80) | 71  (47-85) | 58  (41-70) | 76  (40-84) | 79.5  (63-90) | 73.5  (60-77) | 55.5  (24-70) | 64  (41-73) | 76  (64-76) | 68  (56-71) |
| Median age at onset of prosopagnosia | 64  (39-79) | 67  (48-80) | 75  (49-90) | 62  (48-72) | 77  (65-90) | 82  (81-92) | 76  (64-79) | 63  (26-77) | 64  (41-74) | 80  (71-82) | 71  (63-76) |
| Median age at neurological examination | 65  (41-79) | 69  (50-84) | 77  (49-90) | 63  (51-77) | 80  (65-90) | 83  (65-92) | 76  (64-81) | 63  (26-77) | 65  (41-74) | 80  (73-82) | 71  (63-76) |
| No. with hallucinations (%) | 7  (14.0%) | 1  (2.0%) | 2  (5.3%) | 0  (0.0%) | 15 (71.4%) | 0  (0.0%) | 2  (20.0%) | 1  (12.5%) | 2  (33.3%) | 0  (0.0%) | 1  (33.3%) |
| No. with delusions (%) | 0  (0.0%) | 1  (2.0%) | 1  (2.6%) | 1  (3.7%) | 7  (33.3%) | 0  (0.0%) | 1  (10.0%) | 0  (0.0%) | 2  (33.3%) | 0  (0.0%) | 0  (0.0%) |
| No. with limb apraxia (%) | 10 (20.0%) | 0  (0.0%) | 4  (10.5%) | 2  (7.4%) | 0  (0.0%) | 0  (0.0%) | 0  (0.0%) | 0  (0.0%) | 2  (33.3%) | 5  (100.0%) | 1  (33.3%) |
| *No. with other visual agnosia (%) | 36  (72.0%) | 3  (6.12%) | 4  (10.5%) | 0  (0.0%) | 2  (9.5%) | 0  (0.0%) | 1  (10.0%) | 0  (0.0%) | 2  (33.3%) | 0  (0.0%) | 1  (33.3%) |
| No. with hemianopia (%) | 14  (28.0%) | 0  (0.0%) | 1  (2.6%) | 0  (0.0%) | 0  (0.0%) | 1  (5.6%) | 0  (0.0%) | 0  (0.0%) | 0  (0.0%) | 1  (20.0%) | 1  (33.3%) |
| No. with neglect (%) | 6  (12.0%) | 0  (0.0%) | 3  (7.9%) | 0  (0.0%) | 1  (4.8%) | 0  (0.0%) | 0  (0.0%) | 0  (0.0%) | 0  (0.0%) | 1  (20.0%) | 1  (33.3%) |
| No. with behavioral changes (%) | 3  (6.0%) | 25 (51.0%) | 4 (10.5%) | 12 (44.4%) | 4  (19.0%) | 4  (22.2%) | 1  (10.0%) | 8  (100.0%) | 0  (0.0%) | 0  (0.0%) | 1  (33.3%) |
| No. with personality changes (%) | 10  (20.0%) | 20 (40.8%) | 6  (15.8%) | 14 (51.9%) | 5  (23.8%) | 3  (16.7%) | 0  (0.0%) | 8  (100.0%) | 2  (33.3%) | 1  (20.0%) | 1  (33.3%) |
| No. with ophthalmologic examinations (%) | 16  (32.0%) | 4  (8.2%) | 7  (18.4%) | 2  (7.4%) | 6  (28.6%) | 7  (22.2%) | 4  (40.0%) | 1  (12.5%) | 1  (16.7%) | 0  (0.0%) | 1  (33.3%) |
| No. completing autopsy (%) | 2  (4.0%) | 4  (8.2%) | 1  (2.6%) | 2  (7.4%) | 1  (4.8%) | 0  (0.0%) | 1  (10.0%) | 2  (25%) | 0  (0.0%) | 0  (0.0%) | 0  (0.0%) |

*Apperceptive agnosia, simultagnosia and motion agnosia; No. = number

ADD = Alzheimer disease dementia; DLB = Dementia with Lewy bodies, HAS = Hippocampal sclerosis of aging; LPA = Logopenic progressive aphasia; PPS = Primary prosopagnosia syndrome; SMD = Semantic dementia, bvFTD = Behavioral frontotemporal dementia, CJD = Creutzfeldt-Jakob disease, CBS = Corticobasal syndrome, DEM NOS = Dementia not otherwise specified

**Supplementary Table 2: Clinical summaries of migraine associated prosopagnosia.**

| Patient | Summary |
| --- | --- |
| 1 | Forty-three yo. male with a h/o HA since age 7, with h/o of resection of cerebellar astrocytoma and RoRx 10 years earlier and radiation induced meningioma presented with acute visual changes in the setting of chronic debilitating headaches, was found to have simultagnosia and prosopagnosia. MRI scan showed a new right occipital lobe stroke. Diagnosed with SMART syndrome. Over time the prosopagnosia improved but persisted. |
| 2 | Fifty-two yo female with a h/o of resection of cerebellar astrocytoma and RoRx 35 years ago with radiation induced meningoma presented with acuate onset visual changes in the setting of headaches. Was found to have simultagnosia, optic ataxia, oculomotor apraxia and prosopagnosia. MRI showed gyriform enhancement of the right occipital lobe. After treatment there was improvement of the enhancement, but persistence of the prosopagnosia. |
| 3 | Sixty-nine yo. male with h/o of two similar prior spells, 2 years, and 7 years earlier, again presented with sudden onset of topographagnosia and prosopagnosia. No speech or language problems at the time. He does not have headaches with the spells. Spells lasted about 3 hrs, after which time the prosopagnosia resolved. Patient on blood thinners for cardiac disease with normal levels. Head CT scans have been negative for strokes or any lesions. Diagnosed with possible migrainous auras. |
| 4 | Fifty-seven yo. male with a history of childhood headaches. As a teenager, headaches worsened and were associated with visual auras. Reported an unusual headache in his 20s where he was temporarily unable to recognize people. Headache lasted for hours after which time the headache and prosopagnosia resolved. |
| 5 | Forty-nine yo. male with a past medical history of attention deficit hyperactive disorder and classic migraines presented for evaluation and treatment of recurrent vertigo and anxiety. Reported a history of reoccurring temporary face blindness, most of his life. |
| 6 | Twenty-four yo female presented for management of migraine headaches which began 10-years ago. Her headaches are associated with nausea, phonophobia, photophobia and osmophobia. With her headaches she experiences episodes of face blindness. At present, these episodes occur daily with her headaches. MRI head scan shows a small thalamic lesion but otherwise negative. |
| 7 | Forty-eight yo. female presents for evaluation of headaches and possible seizures. Headaches began 15 years ago after right middle cerebral artery aneurysm rupture and surgical evacuation of the hematoma. Since then, she has had daily headaches associated with tingling of the left side of her lip, as well as marching tingling down her left arm. She also describes loss of facial recognition. No further details are provided. CT head shows focal area of encephalomalacia in the right anterior middle cerebral artery distribution. |

SMART = Stroke-like migraine attacks after radiation therapy

**Supplementary Table 3: Pathological diagnoses in 13 cases with neurodegenerative prosopagnosia**

| **Case** | **Age at death** | **Clinical Diagnosis** | **Alzheimer**  **Disease** | **FTLD-tau** | **FTLD-TDP**  **type** | **HpScl** | **Lewy bodies disease** |
| --- | --- | --- | --- | --- | --- | --- | --- |
| 1 | 85 | Alzheimer’s disease dementia | A3B3C3 | - | Type A | Yes | Diffuse Lewy bodies |
| 2 | 80 | Behavioral variant FTD | - | Diffuse AGD | - | - | - |
| 3 | 61 | Behavioral variant FTD | - | - | Type A | Yes | - |
| 4 | 91 | Dementia with Lewy bodies | - | - | - | - | Limbic Lewy bodies |
| 5 | 84 | Logopenic progressive aphasia | A3B3C3 | - | Type A | - | - |
| 6 | 68 | Posterior cortical atrophy | A3B3C3 | - | Type A | Yes | Diffuse Lewy bodies |
| 7 | 74 | Posterior cortical atrophy | A3B3C3 | - | - | - | Amygdala only Lewy bodies |
| 8 | 86 | Primary prosopagnosia syndrome | - | Diffuse AGD | Type A | Yes | - |
| 9 | 73 | Primary prosopagnosia syndrome | - | - | Type C | Yes | - |
| 10 | 61 | Primary prosopagnosia syndrome | - | - | Type C | - | - |
| 11 | 81 | Primary prosopagnosia syndrome |  | - | Type C | - | - |
| 12 | 71 | Semantic dementia | - | Pick’s disease | - | - | - |
| 13 | 61 | Semantic dementia | - | Globular glial tauopathy | - | - | - |

AD = Alzheimer disease; AGD = Argyrophilic grains disease; HpScl = Hippocampal sclerosis; A=Thal phase: B=Braak NFT stage: C=CERAD
